# The Neurodevelopmental Perspective of Surgical Necrotizing Enterocolitis: The Role of the Gut-Brain Axis

**DOI:** 10.1155/2018/7456857

**Published:** 2018-03-11

**Authors:** Chariton Moschopoulos, Panagiotis Kratimenos, Ioannis Koutroulis, Bhairav V. Shah, Anja Mowes, Vineet Bhandari

**Affiliations:** ^1^Department of Pediatrics, Flushing Hospital Medical Center, SUNY-Stonybrook School of Medicine, Flushing, NY, USA; ^2^Division of Neonatology and Center for Research in Neuroscience, Children's National Medical Center, George Washington University School of Medicine, Washington, DC, USA; ^3^Department of Emergency Medicine, Children's National Medical Center, George Washington University School of Medicine, Washington, DC, USA; ^4^Division of Pediatric Surgery, Palmetto Health Children's Hospital, University of South Carolina School of Medicine, Columbia, SC, USA; ^5^St. Christopher's Hospital for Children, Drexel University College of Medicine, Philadelphia, PA, USA

## Abstract

This state-of-the-art review article aims to highlight the most recent evidence about the therapeutic options of surgical necrotizing enterocolitis, focusing on the molecular basis of the gut-brain axis in relevance to the neurodevelopmental outcomes of primary peritoneal drainage and primary laparotomy. Current evidence favors primary laparotomy over primary peritoneal drainage as regards neurodevelopment in the surgical treatment of necrotizing enterocolitis. The added exposure to inhalational anesthesia in infants undergoing primary laparotomy is an additional confounding variable but requires further study. The concept of the gut-brain axis suggests that bowel injury initiates systemic inflammation potentially affecting the developing central nervous system. Signals about microbes in the gut are transduced to the brain and the limbic system via the enteric nervous system, autonomic nervous system, and hypothalamic-pituitary axis. Preterm infants with necrotizing enterocolitis have significant differences in the diversity of the microbiome compared with preterm controls. The gut bacterial flora changes remarkably prior to the onset of necrotizing enterocolitis with a predominance of pathogenic organisms. The type of initial surgical approach correlates with the length of functional gut and microbiome equilibrium influencing brain development and function through the gut-brain axis. Existing data favor patients who were treated with primary laparotomy over those who underwent primary peritoneal drainage in terms of neurodevelopmental outcomes. We propose that this is due to the sustained injurious effect of the remaining diseased and necrotic bowel on the developing newborn brain, in patients treated with primary peritoneal drainage, through the gut-brain axis and probably not due to the procedure itself.

## 1. Introduction

Necrotizing enterocolitis (NEC) is a devastating disease of mainly premature neonates and the most common gastrointestinal emergency in the neonatal intensive care unit (NICU). The overall incidence of NEC is about 1 in 1000 live births [1]. More than 85% of all NEC cases occur in very premature (<32 weeks of postmenstrual age) and particularly in the extremely low birth weight (ELBW) neonates [2]. NEC is characterized by inflammation and ischemic necrosis of the intestinal mucosa as well as by invasion of enteric gas-forming organisms into the intestinal wall. The population of neonates at risk for developing NEC has increased due to recent advances in neonatal care allowing for survival of a greater number of extremely premature neonates [3, 4].

The two main therapeutic options in surgical NEC are primary laparotomy (PL) and primary peritoneal drainage (PPD). Both techniques are associated with significant mortality and morbidity; however, it remains unclear which should be the preferred method. Besides saving the maximum bowel length/surface area, neurologic sequelae is a major concern and is thought to be associated with the level of systemic inflammatory response impacting on the patient's nervous system.

The purpose of this article is to review the existing evidence for the treatment of surgical NEC comparing PPD to PL as the initial approach, focusing mainly on the neurodevelopmental outcomes. The treatment, prognosis, and neurodevelopmental outcomes will be outlined first. The pathobiology of NEC and gut-brain axis (GBA) with proposed molecular pathways will be discussed next, in depth.

### 1.1. Treatment and Prognosis

The treatment of medical NEC focuses on intensive supportive care along with antimicrobial therapy, discontinuation of feeding with initiation of parenteral nutrition, and expectant management [5]. Therapeutic management of surgical NEC with intestinal perforation may range from peritoneal drain placement to multiple laparotomies with or without ostomy creation [6, 7]. The modality chosen is heavily reliant on patient's stability as well as the surgeon's experience with each approach. It remains unclear which procedure is associated with the optimal outcomes for the patients.

**Figure 1:**
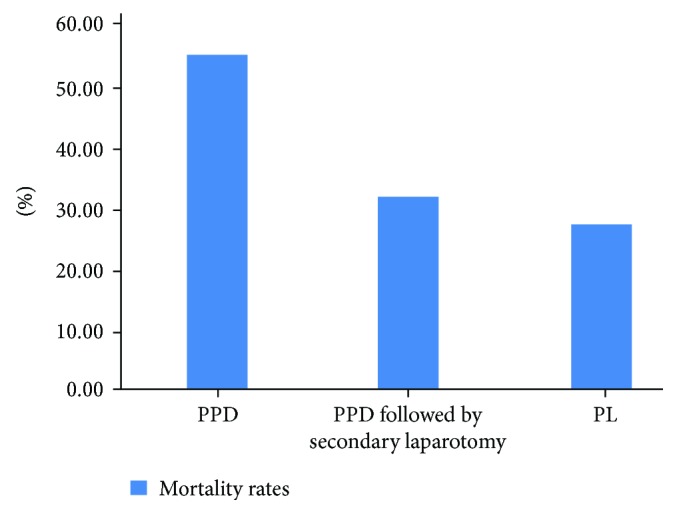
Mean mortality rates in the primary peritoneal drainage only, primary peritoneal drainage followed by secondary laparotomy, and primary laparotomy surgical approaches in preterm neonates with necrotizing enterocolitis in three different studies. In-hospital and overall mortality rates were included [8–10] (*n* = 194,735).

According to the limited existing data, mainly from retrospective studies, PPD alone is associated with increased overall mortality compared with PPD followed by secondary laparotomy or PL. However, this treatment may have been applied to neonates with the highest degree of overall illness (Figure 1) [8–10]. Two separate meta-analyses additionally showed increased mortality rates in PPD compared to PL [11, 12], but no statistically significant difference was found in the meta-analysis of Rao et al. [13].

Rao et al. used the standards of the Cochrane Neonatal Review Group in the interpretation of the data [13]. The authors suggested that the significantly prolonged time to full enteral feeds in the PPD group may be explained by the continued presence of necrotic gut and the associated inflammation [13]. We believe that the sustained injurious effect of the remaining diseased and necrotic bowel may influence the newborn brain through the GBA. Therefore, it is imperative to review the current literature concerning the neurodevelopmental outcomes in medical and surgical NEC, the pathobiology of NEC, and the molecular basis of GBA.

### 1.2. Neurodevelopmental Outcomes

**Figure 2:**
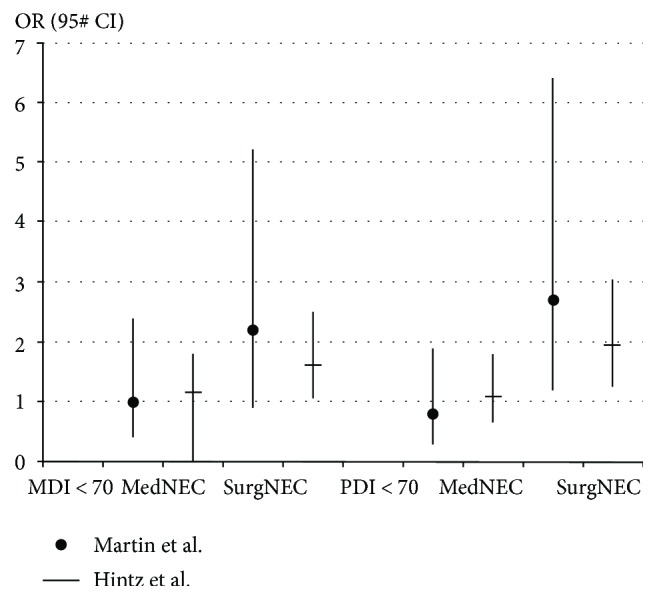
Adjusted odds ratio in two studies for mental developmental index < 70 and psychomotor developmental index < 70 in surgical and medical necrotizing enterocolitis [16, 17]. Patients with medical necrotizing enterocolitis have equal possibility for mental developmental index or psychomotor developmental index < 70 compared to the control group without necrotizing enterocolitis. Patients with surgical necrotizing enterocolitis were more likely to have mental developmental index or psychomotor developmental index < 70 compared to the control patients (*n*1 = 1155, *n*2 = 2948).

Several studies have shown significant neurodevelopmental compromise among survivors with NEC [14, 15]. Neonates with NEC that are managed surgically may have a higher incidence of neurodevelopmental dysfunction compared with neonates that are treated medically only [16–19] (Figure 2). Merhar et al. reported that preterm infants with surgical NEC/spontaneous intestinal perforation (SIP) had more severe brain injury on brain magnetic resonance imaging (MRI) at term compared with infants with medical NEC [20].

In regard to the impact on neurodevelopment, Blakely et al. have conducted the only prospective, multicenter cohort study evaluating neurodevelopmental outcomes at 18 to 22 months in ELBW neonates with NEC or SIP that were treated surgically with either PPD or PL. PL appears to be associated with better neurodevelopmental outcomes, and this may be related to lower rates for the combined outcome of mortality or neurodevelopmental impairment (NDI) compared to those who underwent PPD [21]. A preoperative diagnosis of NEC (versus SIP) was associated with adverse neonatal outcomes (death and death or prolonged total PN) but not with NDI. There was no statistical significant difference in the combined outcome of death and NDI or NDI alone between infants with NEC and SIP [21]. Therefore, the better neurodevelopmental outcome in the PL group was not affected by including both NEC and SIP cases in the population of the study. However, since the pathophysiology of NEC and SIP is different, it is important to launch a large multicenter clinical trial that includes only NEC cases in order to provide the clearest possible NDI outcome between the two surgical approaches in NEC. The risk of NDI was similarly increased among infants with surgical NEC and SIP in a retrospective study [22]. On the other hand, there is existing evidence supporting worse neurodevelopmental outcomes in neonates with intestinal perforation caused by NEC, as compared with SIP [23]. As a result, the better neurodevelopmental outcome of PL may be more relevant for NEC, rather than SIP cases.

Roze et al. concluded that neonates with NEC treated with PL and enterostomy are associated with worse neurodevelopmental outcomes by the age of 6–13 years compared to neonates that received PL and primary anastomosis [24]. Major surgery including laparotomy in VLBW infants is associated with worse neurodevelopmental outcomes compared with infants who underwent minor surgery including peritoneal drainage. The role of general anesthesia is implicated but remains unproven [25]. Increased neuroapoptosis and subsequent neurocognitive or behavioral deficits were induced from the administration of general anesthetic agents to developing animals [26, 27]. However, spinal anesthesia did not produce increased neuroapoptosis in developing rats [28].

The Victorian Infant Collaborative Study Group included in their study extremely preterm or ELBW infants who underwent surgery and required general anesthesia during their primary hospitalization. They were assessed for sensorineural impairments at 5 years of age. The overall rate of sensorineural disability was significantly higher in children who had been operated on compared with those who had not [29]. Filan et al. included in their study preterm infants that were categorized into either a nonsurgical group or a surgical group. After adjustment for birth gestation, BW z-scores, sex, and duration of intermittent positive pressure ventilation, there was no difference in the white matter injury and mental developmental index (MDI) at 2 years [30].

### 1.3. Pathobiology of NEC

The pathogenesis of NEC is multifactorial and likely secondary to immune responses to intestinal microbiota by the premature intestinal tract, leading to inflammation and injury [31]. Gut microflora is different between preterm and full-term infants with a paucity of commensal bacteria at early gestational age (GA) [32]. Nonphysiologic initial microbial colonization of the premature gut and early dysbiosis is strongly involved in the pathogenesis of NEC [33, 34]. Preterm infants with NEC have significant differences in the diversity of the microbiome compared with preterm controls [35]. The gut bacterial flora changes remarkably prior to the onset of NEC with a predominance of pathogenic organisms [35–37]. Vaginal or cesarean delivery seems to have an influence on the diversity and function of the infant's microbiota [38]. Single nucleotide polymorphisms in several genes, including the interleukin- (IL-) 4 receptor [39], IL-18 [40], and the nuclear factor kappa B1 (NF-*κ*B1) variant [41], are associated with the severity of NEC. The binding of NF-*κ*B to the inhibitor kappa B (I-*κ*B) contributes to the tolerance of the gastrointestinal tract to certain commensal bacteria [42]. Once NF-*κ*B dissociates from I-*κ*B, it is able to enter the nucleus, where it controls the transcription of inflammatory mediators [43, 44]. This dissociation is mediated by the toll-like receptor 4 (TLR4). TLR4 is overexpressed in the gut epithelial cells of premature neonates. Recognition of lipopolysaccharide (LPS) by TLR4 is associated with an increase in the expression of NF-*κ*B and proinflammatory mediators [45]. Recently, a study noted a novel association between a hypomorphic variant in an autophagy gene (ATG16L1) and NEC in premature infants [46]. Decreased autophagy arising from genetic variants may confer protection against NEC [46]. Injury to Paneth cells (PCs) contributes to the pathogenesis of NEC. These specialized epithelia protect intestinal stem cells from pathogens stimulating their differentiation, stabilizing the intestinal microbiota, and repairing the gut [47]. Destruction of PCs can lead to bacterial invasion and severe inflammation [47]. Brain TLR4 activation by LPS entering the systemic circulation after enteric bacterial translocation is another potential role of this receptor in the model of GBA [48].

### 1.4. Molecular Basis of GBA

**Figure 3:**
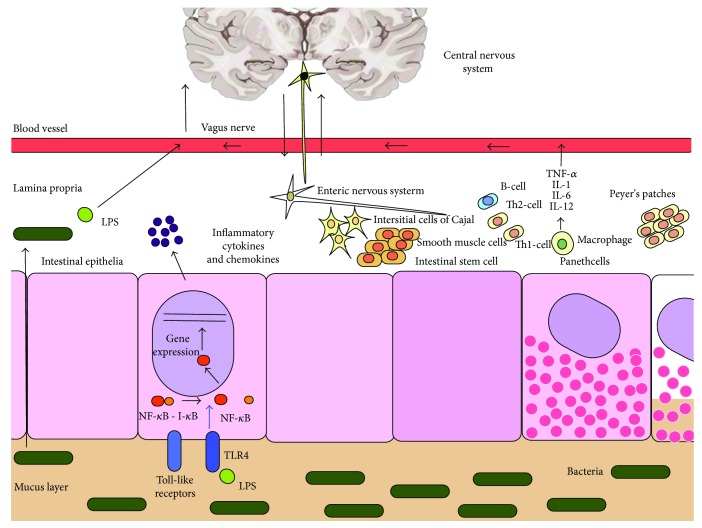
Proposed mechanism in the pathobiology of the gut-brain axis. The vagus nerve contributes to the bidirectional communication between the enteric nervous system and the limbic system inside the central nervous system. Gut microbes may influence brain development and function through the enteric nervous system. Brain TLR4 activation by LPS entering the systemic circulation is a potential role of this receptor in the model of gut-brain axis [34].

It has been proposed that bowel injury might initiate systemic inflammation potentially affecting the developing central nervous system (CNS) [16]. The concept of a GBA has existed for more than 3 decades [49]. The Human Microbiome Project aims to reveal opportunities to improve human health through monitoring or manipulation of the human microbiome [50] and has been associated with recent and rapid advances in GBA-related research [51]. The data on the GBA is primarily associative, and more work needs to be done in order to support causality. The hypothalamic-pituitary axis (HPA), the autonomic nervous system (ANS), and the CNS are integrated peripheral components of the GBA [52]. The sympathetic and enteric nervous systems are mainly responsible for the interaction between the peripheral and the central components of the GBA in a bidirectional model [53, 54]. The limbic system, and specifically the hippocampus, is the locus inside the CNS that is mainly responsible for gut control as shown in studies in neonatal mice [55–57]. Neurobehavioral disorders during childhood seem to be associated with hippocampal injury in preterm infants [58]. The enteric nervous system (ENS) residing within the intestinal wall communicates with the CNS through the vagus nerve, root, and nodose ganglia [54, 59]. Gut microbes might influence brain development and function through the ENS [53]. Alterations in behavior and cognition are associated with the differential microbial composition, since some gut-microbial products can act as “neuro-nucleo-modulins” and thereby affect the epigenetic landscape of their host's brain cells which, in turn, has effects on host behavior [60]. Signals about microbes in the gut are transduced to the brain and the limbic system via the ENS, ANS, and HPA [61]. The afferent and efferent vagus nerves play an important role in this bidirectional communication [62, 63]. The efferent vagus nerve is associated with the regulation of cytokines in the gut leading to inflammation and loss of the intestinal epithelial barrier function allowing bacterial invasion [63, 64]. The function of dendritic and T cells that are located throughout the intestinal wall and can regulate an inflammatory or anti-inflammatory response is modulated by neuropeptides such as vasoactive intestinal polypeptide and norepinephrine [65] (Figure 3).

Gut microbes and probiotic bacteria influence brain development and function [53, 66–68]. Trials of probiotics in neonates showed a reduction in the relative risk for NEC [66–68] that may be due to the release of inhibitors of tumor necrosis factor-alpha (TNF-*α*) and NF-*κ*B from the probiotic bacteria [69, 70]. Blocking the transport of damaging biomolecules via the GBA is another potential mechanism favoring the use of probiotics in the prevention of brain injury [71, 72].

Experimental models of intestinal injury have shown that alteration in gut microbiota may cause brain injury and inflammation. Induced precocious gastrointestinal barrier maturation caused low-grade systemic inflammation and altered short-chain fatty acid utilization in the brain in suckling rats [73]. Changes in neural tissue microstructure, particularly in white matter structural integrity, were found to be associated with diet-dependent changes in gut microbiome populations [74]. Imbalances of the HPA axis caused by intestinal microbes resulted in an anxiety-like behavioral phenotype in mice [75]. The antidepressant effects of two enantiomers of ketamine in chronic social defeat stress model of depression in mice may be partly mediated by the restoration of the gut microbiota [76].

Intestinal microbiota is affected by the administration of antibiotics. There is currently a high degree of variability in the antibiotic regimen for the treatment of NEC, with no regimen appearing superior over another [77]. Prolonged administration of antibiotics is related to adverse neonatal outcomes [78]. Antibiotics with anaerobic coverage, such as clindamycin, are associated with the development of intestinal strictures [79, 80]. Bowel structural changes, such as the development of intestinal strictures, may predispose to alterations in the gut microbiome population. In addition, studies have suggested that an overall reduction in the diversity of microbiome as seen following prolonged antimicrobial therapy is associated with NEC [81, 82]. This finding can be explained by the direct influence of the antibiotic administration in the equilibrium of the intestinal microbiota. Changes in the microbiota population may also initiate systemic inflammation inside the CNS through the GBA. The type of initial surgical management of NEC has an impact on the length of functional gut. The diseased and necrotic bowel is present in patients treated with PPD, compared to those treated with PL, and may lead to brain injury and inflammation through the GBA. Ongoing antibiotic administration may contribute to further CNS inflammation in patients treated with PPD.

In summary, predicting outcomes in neonates with severe NEC is challenging, due to the multiple coexisting comorbidities of the premature patients. Currently, from the limited existing body of evidence, it appears that medical NEC is associated with more favorable neurodevelopment compared with surgical NEC [16–19] and there is significant NDI among survivors [14, 15]. Primary anastomosis in PL is associated with better neurodevelopmental outcomes than stoma formation [24]. PPD alone is associated with increased overall mortality compared with PPD followed by secondary laparotomy or PL [8–10].

The only, to date, prospective cohort addressing the neurodevelopment following PPD versus PL including data from 16 clinical centers within the National Institute of Child Health and Human Development Neonatal Research Network showed that PL is associated with better neurodevelopmental outcomes and is related to lower rates for the combined outcome of mortality or NDI compared with PPD in patients with NEC or SIP [21].

We believe that PL is associated with more optimal neurodevelopment because it prevents the sustained injurious effect of the remaining diseased and necrotic bowel on the newborn brain through the GBA in patients with NEC.

## 2. Conclusion

The fulminant nature of advanced NEC in fragile neonates is a limiting factor in assessing the neurodevelopment outcomes following PPD versus PL approaches. The added exposure to inhalational anesthesia in infants undergoing PL is an additional confounding variable, but requires further study. In regard to neurodevelopment, it appears that existing data favor patients who were treated with PL over those who underwent PPD. We propose that this is due to the sustained injurious effect of the remaining diseased and necrotic bowel on the developing newborn brain, in patients treated with PPD, through the GBA and probably not due to the procedure itself.

## Abbreviations

NEC: Necrotizing enterocolitis
SIP: Spontaneous intestinal perforation
ELBW: Extremely low birth weight
VLBW: Very low birth weight
PPD: Primary peritoneal drainage
PL: Primary laparotomy
TPN: Total parenteral nutrition
GBA: Gut-brain axis
NDI: Neurodevelopmental impairment
CNS: Central nervous system
ENS: Enteric nervous system
HPA: Hypothalamic-pituitary axis
OR: Odds ratio
RR: Relative risk
CI: Confidence interval
MDI: Mental developmental index
PDI: Psychomotor developmental index
RCT: Randomized clinical trial.
